# Clinical application of MRI-respiratory gating technology in the evaluation of children with obstructive sleep apnea hypopnea syndrome: Erratum

**DOI:** 10.1097/MD.0000000000010280

**Published:** 2018-03-30

In the article, “Clinical application of MRI-respiratory gating technology in the evaluation of children with obstructive sleep apnea hypopnea syndrome”,^[1]^ which appeared in Volume 97, Issue 4 of *Medicine*, Dr. Linping Hu's name appeared incorrectly as Linpin Hu.

Figures 1 and 2 were reversed and should appear as:

**Figure 1:**
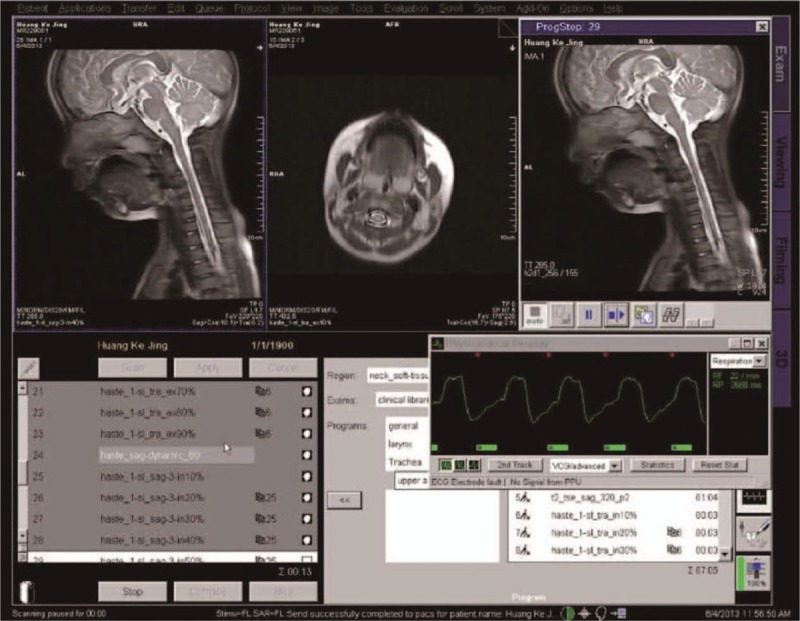
Panel for setting the parameters of the MRI-respiratory gating technology.

**Figure 2:**
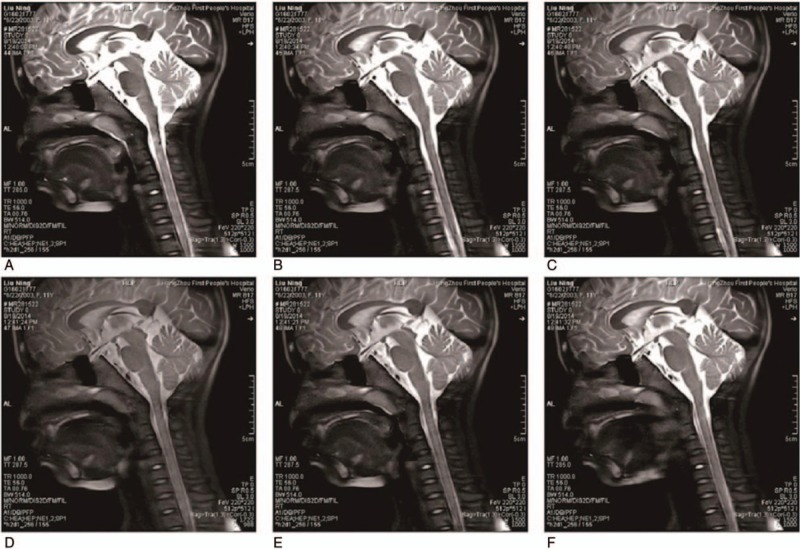
Median sagittal MRI of the upper airway at the 6 respiratory phases. (A–C) Sagittal MR images of the upper airway at 10%, 50%,and 90% of the inspiration phases. (D–F) Sagittal MRI of the upper airway at 10%, 50%, and 90% of the expiration phases.
